# Is the subcallosal medial prefrontal cortex a common site of atrophy in Alzheimer's disease and frontotemporal lobar degeneration?

**DOI:** 10.3389/fnagi.2012.00032

**Published:** 2012-11-23

**Authors:** Olof Lindberg, Eric Westman, Sari Karlsson, Per Östberg, Leif A. Svensson, Andrew Simmons, Lars-Olof Wahlund

**Affiliations:** ^1^Department of Neurobiology, Care Sciences and Society, Karolinska InstitutetStockholm, Sweden; ^2^Aging Research Centre, Karolinska InstitutetStockholm, Sweden; ^3^Division of Speech and Language Pathology, Department of Clinical Science, Intervention and Technology, Karolinska InstitutetStockholm, Sweden; ^4^Department of Medical Physics, Karolinska University HospitalStockholm, Sweden; ^5^Institute of Psychiatry, King's College LondonLondon, UK; ^6^NIHR Biomedical Research Centre for Mental HealthLondon, UK

**Keywords:** Alzheimer's disease, frontotemporal dementia, subcallosal medial prefrontal cortex, MRI

## Abstract

Regions affected late in neurodegenerative disease are thought to be anatomically connected to regions affected earlier. The subcallosal medial prefrontal cortex (SMPC) has connections with the dorsolateral prefrontal cortex (DLPFC), orbitofrontal cortex (OFC), and hippocampus (HC), which are regions that may become atrophic in frontotemporal lobar degeneration (FTLD) and Alzheimer's disease (AD). We hypothesized that the SMPC is a common site of frontal atrophy in the FTLD subtypes and in AD. The volume of the SMPC, DLPFC, OFC, HC, and entorhinal cortex (EC) were manually delineated for 12 subjects with frontotemporal dementia (FTD), 13 with semantic dementia (SD), 9 with progressive nonfluent aphasia (PNFA), 10 AD cases, and 13 controls. Results revealed significant volume loss in the left SMPC in FTD, SD, and PNFA, while the right SMPC was also atrophied in SD and FTD. In AD a non significant tendency of volume loss in the left SMPC was found (*p* = 0.08), with no volume loss on the right side. Results indicated that volume loss reflected the degree of brain connectivity. In SD and AD temporal regions displayed most atrophy. Among the frontal regions, the SMPC (which receives the strongest temporal projections) demonstrated most volume loss, the OFC (which receives less temporal projections) less volume loss, while the DLPFC (which is at multisynaptic distance from the temporal regions) demonstrated no volume loss. In PNFA, the left SMPC was atrophic, possibly reflecting progression from the left anterior insula, while FTD patients may have had SMPC atrophy at the initial stages of the disease. Atrophy of the SMPC may thus be affected by either initial temporal or initial frontal atrophy, making it a common site of frontal atrophy in the dementia subtypes investigated.

## Introduction

In recent years the view that regional atrophy in dementia results from damage to particular brain networks has received increased attention. This view not only relates regional damage to impairment of mental functions, dependent on different brain networks, but also allows inference as to how and where atrophy develops during the course of disease. The assumption is that “later-affected regions bear known anatomical connections with the sites of earlier injury” (Seeley et al., 2009, p. 42). We will refer to this as the “connection hypothesis.” The basic mechanism of the connection hypothesis has previously been demonstrated in monkey brain (Woolsey, 1947; Jones and Powell, 1970) and in neuropathological postmortem studies of Alzheimer's disease (AD) (Pearson et al., 1985; De Lacoste and White, 1993).

The underlying mechanism of the connection hypothesis is that molecular pathologies such as β-amyloid, tau, α-synuclein, and TDP-43 aggregate and progress through specific anatomical connections or brain networks (Seeley et al., 2009; Raj et al., 2012). Support for this assumption in both AD and FTLD has been presented in a large study by Seeley et al. (2009). In another study a model of brain connectivity was derived from whole brain tractography on diffusion MRI scans on 14 healthy young controls. On the basis of the strength of connectivity found in this model several networks were proposed that in subsequent analysis was shown to correspond well with Seeley's assumption of network-specific progression of atrophy in FTLD and AD (Raj et al., 2012).

AD and semantic dementia (SD), which is a subtype of frontotemporal lobar degeneration (FTLD), are characterized by severe hippocampal and temporal lobe pathology while the frontal lobes are initially largely spared. This could be interpreted as support for the connection hypothesis because most frontal regions are at a “multisynaptic” distance from the temporal regions first affected. Most regions in the frontal lobe interact with the hippocampus (HC) through the cingulate and posterior parahippocampal gyri and entorhinal cortices (Goldman-Rakic et al., 1984). Disease processes emanating from the temporal cortex must thus progress through a number of synaptic connections to reach the frontal parts of the brain.

Two regions in the frontal lobe, the subcallosal medial prefrontal cortex (SMPC), and to a lesser extent the orbitofrontal cortex (OFC), are exceptions to the multisynaptic communication pattern described above. Studies with retrograde and anterograde tracers on the rhesus monkey brain show that these frontal regions receive direct projections from the hippocampal formation (Goldman-Rakic et al., 1984; Barbas and Blatt, 1995; Carmichael and Price, 1996). Such direct connections also exist in humans (Kahn et al., 2008). The direct projections from the HC to the SMPC and ORB originate mainly from the CA1 field and the subiculum, and contrary to many brain connections, they are strictly ipsilateral and unidirectional (Barbas and Blatt, 1995; Laroche et al., 2000).

Monkey studies suggest that the entorhinal cortex (EC), heavily affected in AD, also projects particularly to the OFC and the SMPC (Ongur and Price, 2000; Munoz and Insausti, 2005; Insausti and Amaral, 2008). There is, however, less convincing evidence for such direct connections between the EC and the SMPC in humans (Kahn et al., 2008).

In addition to the temporal regions, the SMPC has reciprocal connections with several regions in the frontal lobe. Brodmann areas (BA) 9 and 46 in the dorsolateral prefrontal cortex (DLPFC) are connected to BA14 in the SMPC (Carmichael and Price, 1996; Ongur and Price, 2000). It has further been demonstrated that some areas of the SMPC (such as BA25) have reciprocal connections with regions in the OFC, as well as some parts of the anterior agranular insula (Carmichael and Price, 1996; Ongur and Price, 2000).

In accordance with the connection hypothesis it can be assumed that the SMPC and the OFC become pathologically involved in dementia characterized by temporal/hippocampal pathology. This has indeed been shown in both SD (Whitwell and Jack, 2005; Schroeter et al., 2007; Rohrer et al., 2009) and AD (Thompson et al., 2007). According to the connection hypothesis, atrophy might also progress to the SMPC from a number of frontal regions. In the behavioral variant of FTLD called frontotemporal dementia (FTD), the OFC becomes atrophic early (Perry et al., 2006), while the left anterior insula may be the first area to display atrophy in progressive nonfluent aphasia (PNFA) (Rohrer et al., 2009).

The hypothesis of the current study is that the SMPC is a common site of frontal atrophy in all types of FTLD as well as AD because of its anatomical connections with regions suggested to be the first sites of atrophy in these diseases.

To study this we compared the degree of atrophy in the SMPC with atrophy in the EC, the HC, the DLPFC and the OFC in AD and in the three subtypes of FTLD (FTD, PNFA, and SD).

## Methods

### Participants

Participants were recruited retrospectively from the Memory Clinic at the Karolinska University Hospital Huddinge, Stockholm, Sweden. All participants went through a standard investigation procedure at the memory clinic. Clinical diagnoses were determined at a multidisciplinary consensus conference with physicians, neuropsychologists, speech-language pathologists, and nurses (Andersson, 2007). FTLD syndromes were diagnosed following international consensus criteria (Neary et al., 1998). Patients with FTLD and AD at different stages of the disease were included. Diagnoses of AD were based on criteria of the ICD-10 International Classification of Diseases, Tenth Revision (ICD-10). The control group (CTL) comprised individuals referred to the memory clinic because of mild subjective forgetfulness in everyday life. Objective cognitive impairment was ruled out through comprehensive neuropsychological assessment (impairment was defined as performance 1.5 SD unit below the age-normal mean on any cognitive test). To further minimize the risk of including participants at the very early stages of neurodegenerative diseases, we included only those participants whose performance did not deteriorate over a minimum of 2-years follow-up. Volumetric MRI data were obtained from 12 FTD, 9 PNFA, 12 SD, and 10 AD patients, as well as 13 CTL subjects.

The study was approved by the Regional Ethical Review Board in Stockholm, Sweden. Demographic and neuropsychological data are presented in Table 1. The dementia groups did not differ in age, but all dementia groups had, as expected, significantly lower Mini-Mental State Examination scores (MMSE; Folstein et al., 1975) than the CTL group.

**Table 1:** **Demographic and neuropsychological characteristics of the investigated groups**.

	**CTL**	**FTD**	**PNFA**	**SD**	**AD**
Number	13	12	9	13	10
Age (sd)	63.0 (7.4)	61.8 (7.4)	63.9 (6.7)	64.2 (7.3)	64.2 (6.8)
Years of disease (sd)	–	2.50 (2.1)^#^	3.5 (1.7)	3.9 (1.9)	3.0 (1.3)
MMSE (sd)	29.2 (0.9)	20.8 (6.1)^*^	16.9 (11.4)^*^	22.6 (6.9)^*^	22.4 (6.5)^*^
Female/male	7/3	9/3	6/3	9/4	7/3

CTL, controls; FTD, frontotemporal dementia; PNFA, progressive nonfluent aphasia; SD, semantic dementia; AD, Alzheimer's Disease; MMSE, mini mental state examination; sd, standard deviation;

* Significantly different from CTL on Kruskal–Wallis test with Mann–Whitney U-test post-hoc.

# Significant longer illness duration than FTD in One-Way analysis of variance with a Tukey post-hoc.

### Image acquisition

T1-weighted MR images were acquired on a 1.5T Magnetom Vision Plus scanner (Siemens Medical Systems, Erlangen, Germany). A 3D magnetization-prepared rapid gradient echo pulse sequence (TR, 11.4 ms; TE, 4.4 ms; TI, 300 ms; flip angle, 10°; NEX, 1) was used to obtain 72 contiguous coronal 2.5-mm sections with a 512 × 144 matrix and a 230-mm FOV.

The original images were subsequently interpolated to a 1 × 1 × 1 mm resolution dataset, on which volumetric analyses were performed. Comprehensive quality control was carried out for all MR images as previously described (Simmons et al., 2009, 2011).

### Cortical parcellation and volumetry

The software program MRIcro (Version 1.37; http://www.mricro.com, http://www.mccauslandcenter.sc.edu/mricro/mricron/) was used for parcellation of the cortex. With this software, an image can be viewed in horizontal, sagittal, and coronal directions simultaneously with a reconstruction of the surface of the brain. Measurements were subsequently performed using the HERMES MultiModality software package (Nuclear Diagnostics, Stockholm, Sweden). Regions of interest were traced manually on contiguous coronal sections. The intracranial volume (ICV) was obtained by using a stereologic point-counting technique comprising manual tracing of the ICV on every fourth slice, following landmarks proposed by Eritaia et al. (2000).

**Figure 1:**
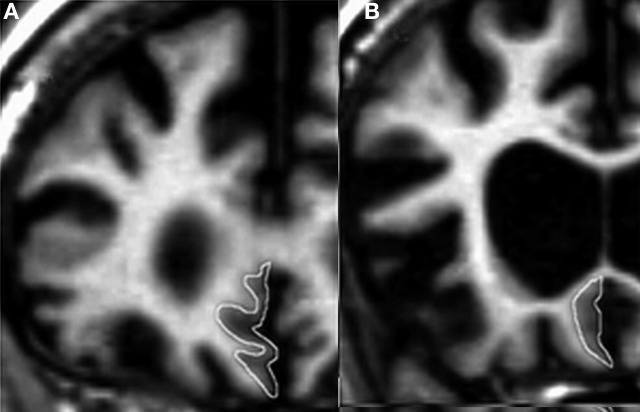
**The subcallosal medial prefrontal cortex (SMPC). (A)** The anterior border of the delineated region. **(B)** The posterior border of the delineated region.

The SMPC was traced in the coronal orientation. The anterior border was defined as the first slice in which the callosal white matter connects the two hemispheres (Figure 1A) and the posterior border was the last slice in which the inferior part of the corpus callosum could be visualized (Figure 1B). Between these landmarks all gray matter on the ventromedial surface was included. Intraclass correlation coefficients (ICCs) were calculated to estimate the reliability of measurements. Tracings of other frontal regions were carried out following Suzuki et al. (2005). For DLPFC we combined the gray matter volume of the superior frontal gyrus with that of the middle frontal gyrus. The reliability of the SMPC measurements was investigated on two occasions and was >0.91 both times. The ICC for other cortical regions has been reported previously (Lindberg et al., 2009), but in short all ICCs were greater than 0.90. All statistical calculations were performed on normalized volume of measured region, derived by dividing the volume of the region by the ICV.

### Statistical analysis

Volumetric data were analyzed by One-Way analysis of variance with a Fisher LSD *post-hoc* test using Statistica 10 (StatSoft, Inc., 2011). All volumetric data were normalized by ICV by the formula volume of region/ICV. A *P* value less than 0.05 was considered significant.

### Correlations between regional volumes

To investigate the relationship between frontal and temporal atrophy with atrophy of the SMPC, Pearson's correlation coefficient (*r*) was calculated for the total normalized volume (left + right side) of each region and the total normalized volume of SMPC.

### Multivariate analysis

Principle component analysis (PCA) is an unsupervised method which does not use *a priori* information about groups for the analysis. The representations of a multivariate data table X, consisting of rows (observations) and columns (variables) as a low-dimensional plane, is an important feature of PCA. Statistically, PCA reduces the dimensionality and complexity of the data by finding lines and planes in the K-dimensional space (*K* = number of variables in the model) that approximates the data in the best way possible in the least squares sense. This provides an overview of the data and allows patterns, trends, and outliers to be observed. It is also possible to view relationships between the observations and the variables. A model usually reduces the K-dimensional space to 2–5 dimensions (Eriksson et al., 2006). The results from PCA are visualized by plotting two components in a scatter plot. Components are vectors in the multivariate space along which groups can be separated. These vectors are dominated by the input variables (x). All the components created by the models are, by definition, orthogonal to each other and span the projection plane of the points. Each point in the scatter plot represents one individual subject. Loadings plots illustrate how the original variables influence the new latent variables (components). The PCA model included all five groups (AD, SD, FTD, PNFA, and CTL) and was created to investigate the constellation of clusters that the program uses to separate dementia patients from controls, not to create a model that effectively separated different variants of dementia.

## Results

### Volumetric analysis

**Figure 2:**
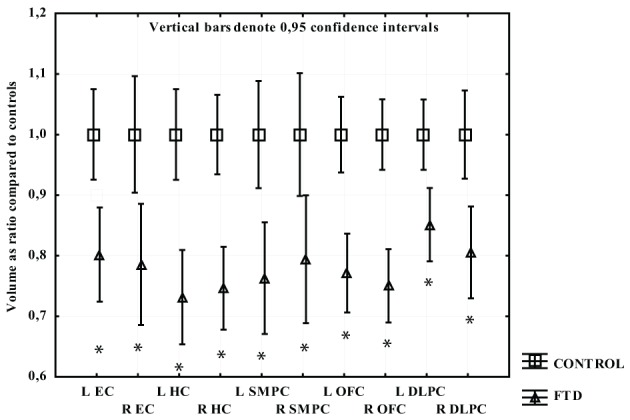
**The volume of measured region in FTD expressed as a ratio of CTL volume**. CTL volume is set to 1. X-axis denotes the included regions: EC, entorhinal cortex; HC, hippocampus; OFC, orbitofrontal cortex; SMPC, subcallosal medial prefrontal cortex; DLPFC, dorsolateral prefrontal cortex. ^*^p < 0.01.

The participants with FTD had significantly smaller gray matter volume than the CTL group in all regions studied. The greatest gray matter loss was found in the OFC, SMPC, and HC with a loss of approximately 25% compared to CTL subjects. The EC and right DLPFC regions had approximately 20% volume loss while left DLPFC had 15% loss relative to CTL subjects (Figure 2).

**Figure 3:**
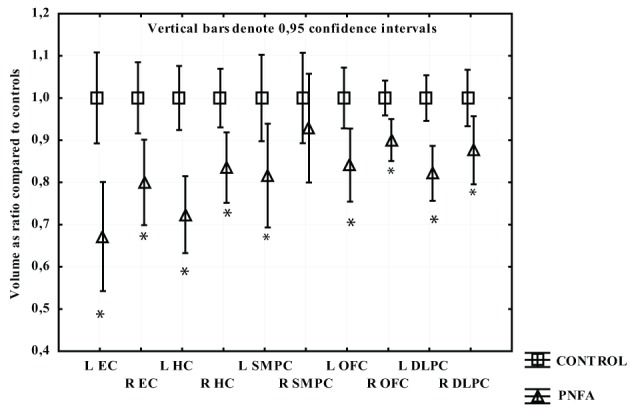
**The volume of measured region in PNFA expressed as a ratio of CTL volume**. CTL volume is set to 1. X-axis denotes the included regions: EC, entorhinal cortex; HC, hippocampus; OFC, orbitofrontal cortex; SMPC, subcallosal medial prefrontal cortex; DLPFC, dorsolateral prefrontal cortex. ^*^p < 0.01.

Participants with PNFA displayed greater volume loss on the left side. All regions had significant volume loss compared to the CTL group except the right SMPC. The left EC displayed the greatest mean gray matter loss compared to CTL, followed by left HC, left SMPC, and left DLPFC (Figure 3).

**Figure 4:**
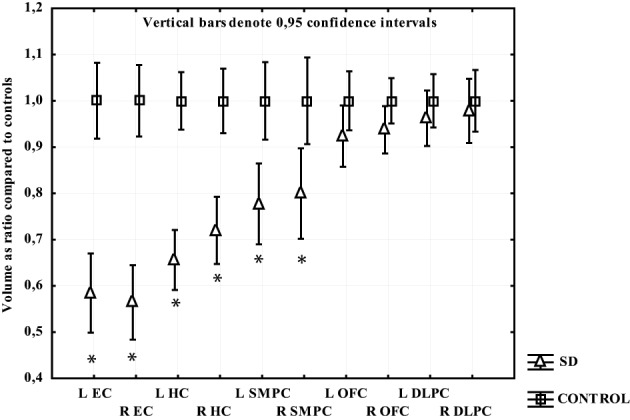
**The volume of measured region in SD expressed as a ratio of CTL volume**. CTL volume is set to 1. X-axis denotes the included regions: EC, entorhinal cortex; HC, hippocampus; OFC, orbitofrontal cortex; SMPC, subcallosal medial prefrontal cortex; DLPFC, dorsolateral prefrontal cortex. ^*^p < 0.01.

All temporal regions and the SMPC displayed significant gray matter loss in participants with SD. The EC displayed most loss (around 40%) followed by HC (30%) and then SMPC (25%). No gray matter loss was found in the OFC or DLPFC (Figure 4).

**Figure 5:**
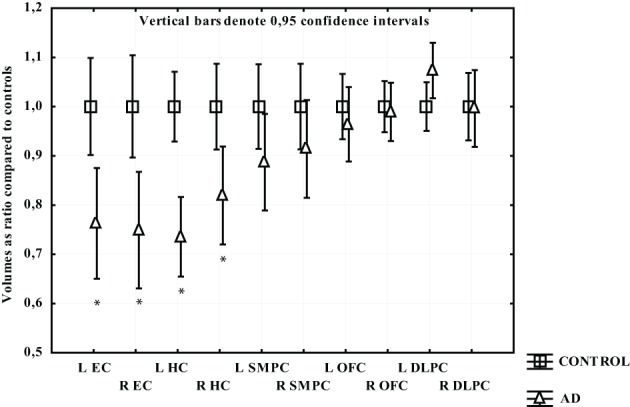
**The volume of measured region in AD expressed as a ratio of CTL volume**. CTL volume is set to 1. X-axis denotes the included regions: EC, entorhinal cortex; HC, hippocampus; OFC, orbitofrontal cortex; SMPC, subcallosal medial prefrontal cortex; DLPFC, dorsolateral prefrontal cortex. ^*^p < 0.01.

In participants with AD only the temporal regions displayed significant gray matter loss. The greatest atrophy was found in EC and left HC (around 25%) followed by right HC (18%). In the left SMPC there was a tendency to volume loss (around 11%; *p* = 0.10), while the right SMPC, left and right OFC and DLPFC did not demonstrate a statistically significant gray matter reduction (Figure 5).

### Principal component analysis (all groups)

**Figure 6:**
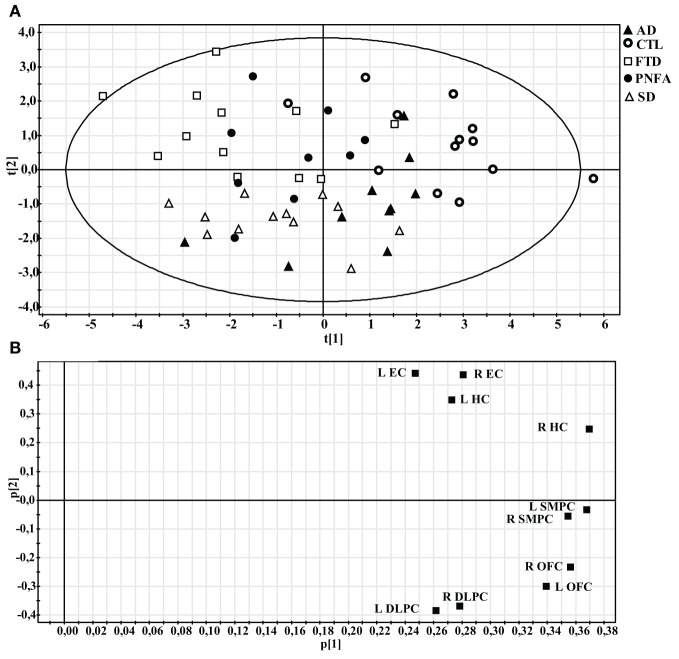
**Principal component analysis (PCA) of all subjects. (A)** Scatter plot illustrating how individuals are distributed along the first two components. **(B)** Loading plot showing the influence of the regional volumes on the first two components.

The PCA model containing all five groups revealed three components, accounting for 70% of the variance of the original data [R^2^(X)] and its cross-validated predictability, Q^2^(X) = 0.48. At the extreme left end of the X-axis on the scatter plot (Figure 6A), we found the dementia cases that displayed most severe frontal and temporal atrophy, while on the right end we found mostly CTL subjects. The loading plot of the PCA may indicate the relationship between frontal and temporal atrophy and the SMPC (Figure 6B). The first component that is plotted along the X-axis can be interpreted as an indicator of general degree of atrophy. The second component (Y-axis) may potentially be interpreted as temporal versus frontal atrophy. Notice that the EC and the HC are plotted relatively close together. The same pattern is observed for DLPFC and OFC.

### Correlations between regional volumes

**Figure 7:**
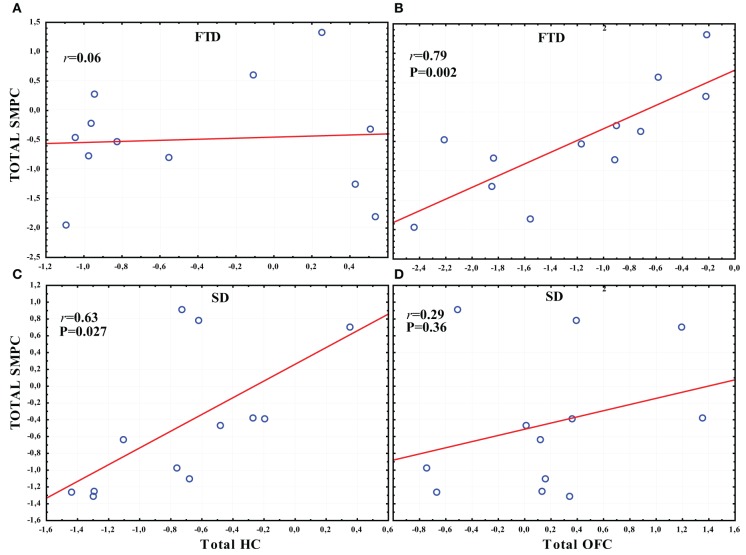
**The correlation between the total volume of SMPC, HC and OFC in SD and FTD. (A)** The correlation between the total volume of SMPC and HC in FTD. **(B)** The correlation between total volume of SMPC and OFC in FTD. **(C)** The correlation between total volume SMPC and HC in SD. **(D)** The correlation between total volume of SMPC and OFC in SD.

In FTD there was a significant correlation between the total normalized volume of OFC and the total normalized volume of SMPC, while no correlation was found between HC and SMPC (Figures 7A,B). The SMPC was also correlated with the total volume of DLPFC (*r* = 0.76, *p* = 0.004).

In SD there was a significant correlation between the total normalized volume of HC and the total normalized volume of SMPC, while no correlation was found between the OFC and SMPC (Figures 7C,D).

The total normalized volume of SMPC was not correlated with any other region in AD or in PNFA.

## Discussion

This study explored the hypothesis that the SMPC may be particularly vulnerable to atrophy in FTLD and AD because of its anatomical connections with frontal and temporal regions that become atrophic in these diseases. In AD and SD, the EC and the HC have been found to be early sites of atrophy (Braak et al., 1996; Rohrer et al., 2009). The SMPC may receive the densest efferent hippocampal projections in the frontal lobe (Barbas and Blatt, 1995). The OFC receives less dense projections (Munoz and Insausti, 2005) while the DLPFC is at a multiple synaptic distance from the HC. This pattern of connectivity is reflected by the volumetric data. The EC and the HC have most volume loss, followed by the SMPC and the OFC, while the DLPFC has no loss of gray matter volume in AD and SD. As discussed in the introduction, there are some differences between man and monkey in the findings concerning connectivity between the EC and the frontal lobe. If there are direct projections from the EC to the SMPC, then the atrophy of the EC could (as in HC) progress directly to the SMPC. Another possibility is that atrophy of the EC progresses to the HC as these regions are reciprocally connected (Pearson et al., 1985; De Lacoste and White, 1993), and from the HC to the SMPC.

In FTD, volumetric analyses revealed that the OFC and the HC was the most atrophic region compared to CTL. The SMPC has, however, almost as much volume loss. One previous study suggests that the OFC may be the first site of atrophy in FTD (Perry et al., 2006). Another study suggests that the so-called paralimbic network (of which the SMPC is part) becomes atrophic first (Seeley et al., 2008). We found a non-significant difference between the degree of atrophy in the OFC and the SMPC, which potentially could support the view that the SMPC is part of a network that becomes affected first in FTD.

In PNFA, left but not right SMPC was atrophic compared to controls. Volume loss in the left SMPC might reflect a progression from initial atrophy in the left anterior insula.

We hypothesized that we also would find atrophy in the SMPC in AD, however only a tendency was found on the left side. The main reason for this was probably the relatively small number of cases included in this dementia group. One reason for not including more AD patients was that we wanted to have approximately the same statistical power for each dementia group. The FTLD group represents almost all patients with this rare diagnosis treated at the memory clinic at Huddinge hospital during a period of 10 years. It should also be noted that the general degree of atrophy was less severe in the AD cases than in the FTLD patients. Another strong indication that HC and SMPC atrophy may be connected is that the ratio of HC volume and SMPC volume is almost identical for AD and SD. Thus the ratio between left HC/Left SMPC is 0.85 in SD and 0.83 in AD, and the ratio between R HC/R SMPC is 0.90 for both SD and AD.

From the discussion above it could be suggested that the development of atrophy in the SMPC may be affected both by frontal as well as temporal atrophy. The results of the PCA may support this finding. As noted in the results section, the EC and the HC as well as the DLPFC and the OFC are closely plotted together in the PCA loading plot. The SMPC is plotted almost in the centre between the EC/HC and the DLPFC/OFC. This could indicate that atrophy of the SMPC may be almost as related to frontal as to temporal atrophy.

This assumption may also be supported by the findings of our correlation analyses. FTD was the dementia subtype that displayed most frontal atrophy (centered in the OFC). In this subtype the total volume of SMPC was correlated with the total volume of OFC, but not with the total volume of HC (Figures 7A,B). SD is the subtype that displayed most temporal atrophy centered on HC and EC. Here the total volume of the SMPC was correlated with the total volume of HC, but not with the total volume of OFC (Figures 7C,D). Thus the volume of SMPC is correlated to a region with severe frontal atrophy in FTD and to a region with severe temporal atrophy in SD.

Atrophy and laterality of atrophy of the SMPC may also be relevant for behavioral and neuropsychiatric alteration in the variants of dementia included in this investigation. The SMPC is the most posterior part of the ventromedial prefrontal cortex (VMPC). The VMPC has for example been associated with the ability to infer other persons' thoughts and feelings often referred to as “theory of mind” (Gregory et al., 2002), the construct of empathy (Shamay-Tsoory, 2010) and the broad concept of “emotional intelligence,” which encompasses a number of social or emotional abilities that enable individuals to smoothly interact in or adapt to a social environment (Bar-On et al., 2003). The right VMPC may be particulary important for certain social abilities, such as theory of mind (Shamay-Tsoory et al., 2005) and empathy (Shamay-Tsoory et al., 2003). Rosen et al. (2005) also found that right SMPC atrophy was associated with disinhibition in dementia, while atrophy of the more anterior parts of the right VMPC was associated with apathy. They have further shown that the right SMPC is important for self-appraisal (the ability to assess one's own abilities) (Rosen et al., 2010).

Our data suggest that the left SMPC may become atrophic in PNFA and AD, while the right side is also involved in FTD and SD. Considering the relative importance of the right side for behavior symptoms (Rosen et al., 2005; Shamay-Tsoory et al., 2005; Rosen et al., 2010) it could be hypothesized that FTD and SD display more frequent alteration of behavior than PNFA and AD. Indeed this has been described in the international consensus criteria for diagnosing the subtypes of FTLD. FTD patients may display “decline in social interpersonal conduct” and SD patients may show “loss of sympathy and empathy” (Neary, 1999). PNFA on the other hand is described as having “early preservation of social skills.” For the diagnosis of AD the American Psychiatric Association ([*DSM-IV-TR*], 2000) does not include deficits in social interaction skills, focusing on memory deficits as a core diagnostic feature, in addition to at least one of the following symptoms: aphasia, apraxia, agnosia or defecits in executive functioning.

The fact that the left SMPC may become more involved in AD may potentially also be explained by the connection hypothesis. Several previous studies have found that hippocampal atrophy at initial stages of AD may be more severe on the left side (Shi et al., 2009). Thus pathology may first progress to the left SMPC/VMPC and then as the disease develops to the right temporal lobe and right SMPC/VMPC. Thus the right VMPC may initially be relatively spared in AD and PNFA which may preserve these patients social interaction skills longer than in SD and FTD.

The most important limitation of this study is the lack of longitudinal data to provide direct evidence for how atrophy develops in the brain. The main point of this work is however that brain connectivity in cross-sectional data may provide important clues as to how and where atrophy may develop during the progression of neurodegenerative diseases.

Another limitation is that only structural 3D images were available in this study. Other MRI-techniques such as diffusion tensor imaging (Catani et al., 2012) or resting state MRI (Yi et al., 2012) could potentially reveal signs of pathology in brain networks before atrophy of regions that belong to these networks become detectable.

A third factor that needs to be considered in the interpretation of our results is the characteristics of our control group who sought consultation at the memory clinic because of subjective feelings of forgetfulness. While objective memory deficits were neither found at baseline investigation nor at follow up (with a minimal interval of 2 years), this does not exclude that these individuals may develop a neurodegenerative disorder later than two years after first examination. Differences between the investigated neurodegenerative disease and controls may thus potentially be even larger if subjects without subjective memory complaints had been used as controls.

## Conclusions

Our finding supports the view that the SMPC, owing to its anatomical connections, may become a common site of frontal pathology in AD and FTLD. This supports the assumption that progression of atrophy in dementia may be predicted on the basis of the anatomical connectivity of the first atrophic region. Knowledge of the regional connectivity of the brain may thus help to predict in which regions atrophy will appear in the progression of neurodegenerative diseases.

## Author contributions

Olof Lindberg and Eric Westman substantially contributed to conception and design, or acquisition of data, or analysis and interpretation of data. Olof Lindberg, Eric Westman, Sari Karlsson, Per Östberg, Andrew Simmons, Leif A. Svensson, Lars-Olof Wahlund to the drafting of the article or revising it critically for important intellectual content.

### Conflict of interest statement

The authors declare that the research was conducted in the absence of any commercial or financial relationships that could be construed as a potential conflict of interest.
